# Efficacy and safety of Ho:YAG Laser Lithotripsy for ureteroscopic removal of proximal and distal ureteral calculi

**DOI:** 10.1186/1471-2490-14-62

**Published:** 2014-08-08

**Authors:** Wael Y Khoder, Markus Bader, Ronald Sroka, Christian Stief, Raphaela Waidelich

**Affiliations:** 1 Department of Urology, University Hospital Grosshadern, Ludwig-Maximilians University, Marchioninistrasse 15, D - 81377 Munich, Germany; 2 Laser Research Laboratory/LIFE Center, Ludwig-Maximilians-University, Munich, Germany

**Keywords:** Ureteral calculi, Ureteroscopy, Holmium-YAG laser, Lithotripsy

## Abstract

**Background:**

Laser lithotripsy is an established endourological modality. Ho:YAG laser have broadened the indications for ureteroscopic stone managements to include larger stone sizes throughout the whole upper urinary tract. Aim of current work is to assess efficacy and safety of Ho:YAG laser lithotripsy during retrograde ureteroscopic management of ureteral calculi in different locations.

**Methods:**

88 patients were treated with ureteroscopic Ho:YAG laser lithotripsy in our institute. Study endpoint was the number of treatments until the patient was stone-free. Patients were classified according to the location of their stones as Group I (distal ureteric stones, 51 patients) and group II (proximal ureteral stones, 37). Group I patients have larger stones as Group II (10.70 mm vs. 8.24 mm, respectively, P = 0.020).

**Results:**

Overall stone free rate for both groups was 95.8%. The mean number of procedures for proximal calculi was 1.1 ± 0.1 (1–3) and for distal calculi was 1.0 ± 0.0. The initial treatment was more successful in patients with distal ureteral calculi (100% vs. 82.40%, respectively, P = 0.008). No significant difference in the stone free rate was noticed after the second laser procedure for stones smaller versus larger than 10 mm (100% versus 94.1%, P = 0.13). Overall complication rate was 7.9% (Clavien II und IIIb). Overall and grade-adjusted complication rates were not dependant on the stone location. No laser induced complications were noticed.

**Conclusions:**

The use of the Ho:YAG laser appears to be an adequate tool to disintegrate ureteral calculi independent of primary location. Combination of the semirigid and flexible ureteroscopes as well as the appropriate endourologic tools could likely improve the stone clearance rates for proximal calculi regardless of stone-size.

## Background

Laser technologies are established standard modalities for application on lithotripsy [1]. The introduction of the Ho:YAG laser have broadened the indications for ureteroscopic stone managements (URS) to include larger stone sizes throughout the whole upper urinary tract [2]. Furthermore, recent developments in the design of ureteroscopy and endoscopic instruments have enabled the URS to replace the open surgery treatments for urinary calculi over the last decade as a minimally invasive modality.

The Nephrolithiasis Guidelines of both the European Association of Urology (EAU) and American Urological Association (AUA) have focused on the changes in ureteral stone managements. According to these guidelines, the extracorporeal shock wave lithotripsy (SWL) and URS remain the two primary treatment modalities for the management of symptomatic ureteral calculi [3]. This was based on their meta-analysis. There was no difference in stone-free rates between SWL and URS after all primary procedures in the proximal ureter (82% versus 81%, respectively). This was dependent on the stone size. For small proximal stones (<10 mm), SWL stone free rate was higher than URS (90% versus 80%, respectively), however, for larger stones (>10 mm), URS stone free rate was superior (79% versus 68%, respectively). Interestingly, URS yields better stone-free rates for distal stones independent of the size (94.5%5 versus 74%, respectively) [3].

We assessed the safety and efficacy of Ho:YAG laser based endoscopic treatment of ureteral calculi whether with a semirigid or flexible ureteroscopy on the improvement of stone-free rates of proximal and distal ureteral stones. In addition, the influence of stone size on the outcome following this ureteroscopic laser lithotripsy was studied for proximal ureteral stones (≤10 mm vs. > 10 mm).

## Methods

Data of 88 patients (25 women and 63 men) who underwent Ho:YAG laser lithotripsy for ureteral stones over two years from June 2011 to June 2013 at our institution were retrospectively reviewed. To omit the experience of the surgeon as a factor in the analysis of the results, two experienced surgeons have operated all the patients of the current study. Ethical approval was obtained from the department of urology, Ludwig-Maximilians-University Ethical Committee.

These were classified into 2 groups according to position of ureteric stones; distal ureteral stones below the pelvic brim (37 patients, group I) and proximal ureteral stones located above the pelvic brim (51, group II).

All patients underwent pre- and post-operative laboratory examinations, including blood pictures, serum biochemistry and urine analysis. Patients with preoperative urinary tract infections received antibiotic therapy according to the culture-specific tests until the culture became negative for infection. Stone size was determined based on the preoperative plain abdominal x-ray or CT examinations. Total linear calculus diameter equals the measured largest linear dimension (transverse or cranio-caudal section) or the sum of the linear measurements of individual calculi for multiple ureteral calculi in one system.

Postoperative evaluation included plain abdominal films taken immediately after treatment before leaving the operating theater. Patients were followed up for a minimum of 3 months with abdominal ultrasonography, plain x-rays or CT scans to exclude obstructive hydronephrosis and to verify the residual stone burden, if present. The study has been approved by our department committee.

Assessment of the procedure outcomes included stone free and complication rates. Stone freedom was defined as pulverization of all calculi to fine dust or fragments not larger than 2 mm in diameter on x-ray imaging at the end of procedure. This was considered to be too small to extract and was liable to pass spontaneously [3]. Patients requiring re-procedures or SWL during follow-up were considered as treatment failures. Operative time was calculated from begin of cystoscopy till the removal of all equipment.

### Laser lithotripsy equipment

Ho:YAG laser system (Auriga XL, Starmedtec, Starnberg, Germany) with laser fiber diameter of 365 μm was used with the semirigid ureteroscopes. Fibers with 220 μm diameter were used for the flexible ureteroscopes to avoid compromising both irrigation flow and the maximal endoscope tip deflection.

### Operative instrumentation and technique

The ureteroscopic manipulations of the upper urinary tract are adequately described [2,4,5], here in brief;

Under general anesthesia, dorsal lithotomy position, and after sterilization and sterile coverings, a diagnostic 22 F cystoscopy was done. Spinal anesthesia are not a common approach in our department as both patients and anesthists advice general anesthesia. This is followed with identification of the ureteral orifice and its cannulation with 0.038-inch hydrophilic guide wire under fluoroscopic guidance. A 16 F transurethral catheter is inserted for drainages to prevent bladder distension during continuous irrigation.

Ureteroscopic procedures were done either with semi-rigid ureteroscopes (diameter from 7 F to 9,5 F) or 7.5 F flexible ureteroscopes implying double active tip deflection mechanism and maintaining a 3.6 F-working channel (Storz Flex *X*2, Storz Tutlingen, Germany). The flexible ureteroscope was positioned via a 12 F ureteral access sheath (Cook Medical Bloomington, Illinois, USA) that was placed over the guide wire. These ureteral access sheaths allow frequent passage of the ureteroscope to the upper urinary tract, enable optimal visualization, maintain low intra-renal pressure and hasten calculi fragments extraction. Ureteric manipulations were aiming to direct laser shock impulses to the middle of stones and their fragments under direct vision to allow fragmentation without ureteric injuries.

Basket stone removal was considered for fragments >2 mm in size after laser fragmentation to achieve samples for stone composition analysis. Staged therapy was considered in case of a bad visibility limiting further access to rest fragments or when remaining stone burden seems larger to still be removed at the same session. Bad visibility was mainly due to macrohematuria as well as stone dust leading to turbidity of fluid media and obscuring vision.

Ureteric stent was placed based on following criteria; prolonged procedures (>60 minutes), large amount of stone debris or evident ureteral edema/trauma and prior insertion of an access sheath. Complications were classified according to the modified Clavien grading system [6].

### Statistical analysis

The Mann–Whitney test was used for comparison of the univariate variables between the two groups. Outcome, complications and operative data were compared using the chi-square and Fisher exact test. Significance was considered as P-value <0.05. All data are expressed as calculated mean ± standard error (SEM). Analysis was done using the computer software SPSS 17.0 for Windows XP.

## Results

No significant difference in median patient’s age between the two groups (53.9 vs. 53.3 year) was found. A part of patient cohorts had undergone previous interventions for calculus disease (39.2% vs. 40% for group I vs. II, respectively). Patients with proximal ureteral stones had larger calculi (median diameter = 10.70 mm) vs. those with distal ones (median 8.24 mm) and were more likely to have DJ-stent at presentation (37.3% vs. 16.2%, respectively). Multiple stones were found in three patients of 1st group versus five patients in 2nd group (Table 1).

**Table 1:** Patients’ and stones’ characteristics: proximal versus distal ureteral calculi in 88 patients treated with ureteroscopic Ho:YAG laser lithotripsy

**Patients’ characteristics**			
**Number of patients**	**Proximal stones 51**	**Distal stones 37**	**P value**
Gender:			
male/female	40/11	23/14	
Age (years)*	53.9 ± 1.8	53.3 ± 3.0	0.829
Stone diameter (mm)*	10.7 ± 0.7	8.2 ± 0.6	0.020
Stone burden (mm^2^)	110.7 ± 17.6	64.4 ± 11.6	0.010
Number of patients with multiple stone burden (%)	3 (5.9)	5 (13.5)	0.225
Number of patients with prior treatments (%)	20 (39.2)	15 (40)	0.905
Number of patients with preoperative double-J stents (%)	19 (37.3)	6 (16.2)	0.032

*mean +/- SD.

Mean operative time was 81.3 ± 4.5 minutes (25–140 minutes) in the first group and 65.7 ± 3.8 minutes (25–120 minutes) in the 2nd group.

Operative characteristics are shown in Table 2. Complete fragmentation during single procedure was achieved in all patients of the 1st group (100%). In the 2nd group, only 42 patients (82.4%) were rendered stone free by a single laser lithotripsy procedure. A staged procedure was necessary in 9 patients due to large residual stone burden and constricted visibility. After a second laser lithotripsy procedure, 48 (94.1%) patients were rendered stone free. Third procedure was necessary in one patient. Three patients (5.8%) underwent SWL as they wished this trial before a third session and it was successful. Ureteral stents were placed at the end of 81 (86.2%) procedures to prevent transient obstruction.

**Table 2:** Operative characteristics, outcome and complications of ureteroscopic Ho:YAG laser lithotripsy in 88 patients: proximal versus distal ureteral calculi

**Number of patients**	**Proximal stones 51**	**Distal stones 37**	**P value**
Ureteroscope number of procedures (%)			
Semirigid	12 (21.1)	37 (100)	< 0.001
Flexible	2 (3.5)		
Combination	43 (75.4)		
Operation time (min)*	81,3 + 4,5	65.7 ± 3.8	0.017
Laser time (sec)*	379.8 + 50.8	154.3 ± 38.1	0.009
Total energy (J)*	2528,8 ± 422.6	1148.5 ± 400.7	0.002
Number of patients with postoperative stent (%)	50 (87.7)	31 (83.8)	0.596
No of intra operative complications (%)	1 (1.8)	2 (5.4)	0.334
No of early post-operative complications (%)	1 (1.8)	3 (8.1)	0.141
Overall SFR per patient	48 (94.1)	37 (100)	0.139
SFR after first treatment per patient (%)	42 (82.4)	37 (100)	0.008
SFR after second treatment per patient (%)	48 (94.1)	---------	---------
No of laser procedures*	1.1 ± 0.1	1.0 ± 0.0	0.027

*mean +/- SD.

The overall stone free rate for both groups was 95.8%. The mean number of all laser procedures was 1.07 ± 0.003, in the proximal ureter 1.1 ± 0.1 and in the distal ureter 1.0 ± 0.0 (P = 0.027). More successful initial treatments were done in the distal ureteral versus proximal calculi (100% vs. 82.40%, respectively).

There were no statistical significant differences in patients’ demographics and operative characteristics when procedures done for proximal ureteral stones were compared as regards stones’ sizes (≤10 mm vs. ≥10 mm in diameter) (Table 3). There was no statistical significant difference between stone free rates after the first versus second laser lithotripsy in 2nd group for stones smaller versus larger than 10 mm (94.1% vs. 100%, respectively, P =0.139) (Table 4).

**Table 3:** Patients and Stones Demographics for 51 patients with proximal ureteric stones exposed to ureteroscopic Ho:YAG laser lithotripsy classified as small (<10 mm) and large (>10 mm) stones

**Patient and stone demographics**			
	**Proximal ≤ 10 mm (n = 31)**	**Proximal > 10 mm (n = 20)**	**P value**
Gender (n)			0.839
Male	24 (77.4)	16 (80)	
Female	7 (22.6)	4 (20)	
Age (years)	53.1 ± 2.2	55.3 ± 3.0	0.549
Mean stone diameter (mm)*	7.4 ± 0.3	15.8 ± 1.1	**<0.001**
Mean stone burden (mm^2^)*	45.2 ± 3.9	212.0 ± 34.0	**<0.001**
No of patients with multiple stone burden (%)	2 (6.5)	1 (5)	0.850
No. of patients with prior treatments (%)	11 (35.5)	9 (45)	0.508
No. of patients with preoperative double-J stents (%)	12 (38.7)	7 (35)	0.800

*mean +/- SD.

**Table 4:** Operative characteristics of 51 patients with proximal ureteric stones exposed to ureteroscopic Ho:YAG laser lithotripsy classified as small (<10 mm) and large (>10 mm) stones

**Operative characteristics**			
	**Proximal ≤ 10 mm (n = 34)**	**Proximal > 10 mm (n = 23)**	**P value**
Ureteroscope			0.778
Semirgid	7 (20.6)	5 (21.7)	
Flexible	2 (5.9)	-	
Combination	25 (73.5)	18 (78.3)	
Mean OR time (min.)	80.7 ± 6.5	82.3 ± 5.9	0.866
Mean laser time	349.5 ± 69.9	434.3 ± 66.9	0.243
Mean total energy (J)	2480.3 ± 585.8	2619.3 ± 547.1	0.483
Postoperative stent (n)	31 (91.2)	19 (82.6)	0.345
Intra operative complications (n)	1 (2.9)	0 (0)	0.431
Post operative complications (n)	1 (2.9)	0 (0)	0.431
Overall SFR per patient	28/31 (90.3)	20/20 (100)	0.163
SFR after first treatment per patient	25/31 (80.6)	17/20 (85)	0.704
SFR after second treatment per patient	28/31 (90.3)	20/20 (100)	0.163
Laser rate	1,1 ± 0,1	1,2 ± 0,1	0.675

Overall complication rate was 7.9%. Perioperative complications were recorded in three and four patients in the 1st versus 2nd group, respectively (Table 2). There were no major perioperative complications noted in this series. All intraoperative complications were classified as clavien grade IIIa (small mucosal laceration without leakage), while all early postoperative complications were Grade II (febrile urinary tract infections). Ureteral mucosal injuries were seen in patients with impacted stones and were managed conservatively resolving within six weeks. Cases of urinary tract infection responded successfully to parenteral antibiotics. Overall and grade-adjusted complication rates did not depend on the stone location. None of these complications was due to laser energy.

Stone analysis was available from 75 patients and revealed calcium oxalate monohydrate (COM) (78.7%), combination of calcium oxalate and phosphate (8.0%), pure calcium phosphate apatite (8%) and >50% uric acid stones (5.3%). Current study showed no influence of stone composition on laser efficacy or complication rates.

The Ho:YAG laser was capable to fragment all stone compositions to acceptable amounts of debris. Mean laser time per patient was longer in the proximal group (379.8 vs. 154.3 seconds). Accordingly, the mean total energy was 2480.3 ± 585.8 vs. 2619.3 ± 547.1 J, respectively (P = 0.483).

## Discussion

Over the last decade, lasers have been increasingly used for intracorporial lithotripsy [1]. Ho:YAG laser has become one of the most widely accepted lasers for this purpose as compared to ultrasonic, pneumatic and other laser devices (e.g. pulsed dye laser, Alexandrite laser and the frequency-double-doubled double-pulse neodymium:yttrium aluminium garnet (FREDDY) laser) [7].

Technical advancements in instruments (semi-rigid ureteroscopy), the advances of the new generation flexible ureteroscopes with greater angles of maximum active tip deflection and improved durability in addition to the introduction of laser lithotripsy with its precise and powerful thermal decomposition mechanism and its excellent safety profile with the ability of delivering laser energy through small, flexible fibers have blazed the trail for fragmentation of stones throughout the upper urinary tract [2,8-11].

The meta-analysis of the EAU/AUA nephrolithiasis guideline panel demonstrated that URS yields significantly greater stone-free rates for the majority of stone stratifications [3]. There were a change in trend in the nephrolithiasis guidelines, which recommended SWL for proximal ureter stones <10 mm (1997), to deeming the ureteroscopic management as a first choice therapy for those stones to improve efficacy and reduce morbidity. However, both guidelines still recommend SWL and ureteroscopy as an option for distal ureteric stones [3,12].

The current study included cohort of patients with ureteral calculi requiring lithotripsy for stone retrieval. We showed a stone-free rate for calculi in the proximal ureter after one and two procedures of 82.4% and 94.1%, respectively. This confirms previous reports in literature about safety and efficacy of ureteroscopic Ho:YAG laser treatment in treating proximal and distal ureteric stones [13-15]. In their meta-analysis, Tiselius et al. reported that re-treatment rates for ureteral stones using URS are lower than SWL (2.2% vs. 12.1%, respectively). This advantage was counter-balanced by the higher need for anesthesia (94.3% vs. 28.3%, respectively) [16].

The current stone-free rate of Ho:YAG laser lithotripsy in the proximal ureter was not significantly different when comparing stones with different sizes (≤10 mm vs. ≥10 mm in diameter or multiple stones) postulating that primary stone size does not influence the efficacy of the procedure. This is comparable to previous reports showing stone free rates of 78% to 96.5% after the first procedure and 88% to 100% after the second laser lithotripsy [3,13-15]. The high success rate for distal ureter stones in the current series after a single procedure is consistent with the literature that shows stone-free rates of 97-100% [10,13,15].

The current mean operative time (81.3 vs. 65.7 minutes, P = 0.017) and laser time (379.8 vs. 154.3 seconds, P = 0.009) was shorter for distal ureteral calculi than for proximal calculi. This could be explained with the larger stone burdens and our effort to completely “melt down” the calculi with laser and the effort to remove as much stone debris as possible leaving no significant fragments. Furthermore, combination of semirigid and flexible ureteroscopy in 75.4% of patients with proximal calculi may add another explanation.

A second procedure was indicated in some of our cases. Many causes attribute to the retrograde stone fragment migration occurring during the treatment of proximal ureteral stones. Gravitational forces, pressurized irrigation, stone retropulsion during lithotripsy, failure to access the calculi or large stone burden may bring the stone out of reach for the semi-rigid ureteroscope [10,13,17,18]. The use of the flexible ureteroscopes in combination with access sheaths provides consistent, reliable and unimpaired access to the upper urinary tract facilitating the treatment of complex proximal calculi and migrated stone fragments and ensuring the clearance of all stone fragments not deemed to pass spontaneously in the same ureteroscopic session [11,19,20]. Clear vision to ease direct access to the targeted stones is essential during ureteroscopic laser lithotripsy. Decreased visibility leads to prolonged operative time and increase the potential risk of injuring the ureter or the flexible ureteroscope.

Generally, routine postoperative ureteral stenting after ureteroscopic laser lithotripsy is still a subject of debate. On one hand, stent-related morbidities like bladder irritation and mild back discomfort during urination were demonstrated to constrain postoperative quality of life. On the other hand, ureteral stenting was thought to prevent postoperative urinary sepsis by avoiding sudden ureteral obstruction by calculus fragments, blood clots or ureteral mucosal edema. The clear indications for stenting include ureteral injury/stricture, solitary kidney, renal insufficiency or large residual stone burden [3]. Furthermore, several prospective randomized controlled trails comparing a non-stented versus stented ureteroscopic lithotripsy reported the same result [21-23]. Ureteral stenting after uncomplicated ureteric procedures is not a routine at our institution. However, a transient ureteral stent is placed in all patients who had ureteral dilatation through the insertion of access sheath, presented with large and/or impacted calculi irrespective of the location. This explains the current high postoperative stenting rates (87.7% vs. 83.8%, respectively).

URS and laser lithotripsy have proved safety even where SWL is likely to fail or contraindicated. Major complications are not common during the procedure [3,24]. Minor intra or postoperative complications were reported in a range from 0 to 13% and consist primarily of pain or urinary tract infection. Ho:YAG laser related complications are as low as 1% [8,10,13,24]. There were no major complications observed in the current series. Further, there was no significant difference of intra and postoperative complications between both groups. Both cases of ureteral mucosal injuries occurred in patients with impacted distal ureteric stones and were successfully managed conservatively within six weeks.

Lastly, any laser is as safe or hazardous as the user and his knowledge and skill, defines how well laser safety measures managed. The appointment of a laser safety officer is mandatory for the safety use of lasers [25-27]. His duties include; review of several protective measures, designation of the appropriate control behaviors according to his facility, approving and maintaining all protective equipment, ensuring that all the staff is properly educated and trained as well as investigating all accidents and incidents. He is recommended to all operations of laser class 3B and 4 in most international regulations [25]. Safety behaviors include e.g. clear communications between personnel in the operating room and isolated field with closed doors, which should be marked with attention light signals. Safety equipment like goggles and fiber fixation tools and laser safety/emergency button should be available [28]. Personnel and surgeons should treat operation equipment and catheters with caution to avoid any parallel damages [28].

The wave length of the Ho:YAG laser varies from 2050 nm to 2100 nm depending on the apparatus. Pulse duration (150-800 μs) depends on the pulse energy, and could be selected only in the latest laser systems. Ho:YAG laser light is mainly absorbed by tissue water, so that it has a mean optical penetration depth of 0,2 mm. The mechanism of its laser induced effect for lithotripsy includes the “Moses” effect, (bubble formation in front of stones) and thermal vaporization of the stone water, thus during expansion fragmentation occurs. This mechanism is accompanied with small fragments and many pulses had to be applied, compared to short pulsed lasers (e.g. Q-switch) which produce large fragments in response to fewer pulses. The later laser lithotripsy is attributed to the shock wave effect of the laser resulting from cavitation-collapse mechanisms [29]. Thus with using Ho:YAG laser pulses, the repulsion effect is reduced compared to the short-pulse laser lithotripsy [29].

There are some limitations of the current study. First, its retrospective character has its inherited limitations. Others include small patients’ series, single institution and the lack of randomization. Larger randomized series may be necessary to confirm the long-term efficacy of this procedure.

## Conclusions

Ho:YAG laser lithotripsy during URS appears to be an adequate tool to disintegrate all kinds of ureteral calculi with low complication rates. The combination of semirigid and flexible ureteroscopes ensures direct access to all migrated, repulsed or floated stone fragments. Destruction of large calculi may be time consuming prolonging operative time and general anesthesia, and could be an indication for staged therapy in some cases.

### Consent

Current study constitute a retrospective evaluation of our collected data about these patients. patients have given a consent for the surgery. All data were anonymised before statistical evaluation by a third party.

## Competing interests

The authors declare that they have no competing interests.

## Authors’ contributions

WYK carried out part of surgical procedures, collection of study data, participated in the statistical evaluations and drafted the manuscript. MB performed rest part of surgeries and participated in the follow up evaluations. RS was responsible for the laser safety and technical issues. CS participated in the design of the study, performed the statistical analysis and supervised the work. RW participated in study design and coordination. All authors read and approved the final manuscript.

## Authors’ information

Wael Khoder and Markus Bader: Shared first authorship.

## Pre-publication history

The pre-publication history for this paper can be accessed here:

http://www.biomedcentral.com/1471-2490/14/62/prepub
